# Myo-Inositol: Pharmacokinetics, Biological Functions, and Therapeutic Potential in Liver Protection: Insights from Preclinical Models

**DOI:** 10.3390/antiox15030297

**Published:** 2026-02-27

**Authors:** Tomasz Antonowski, Adam Osowski, Joanna Wojtkiewicz

**Affiliations:** Department of Human Physiology and Pathophysiology, School of Medicine, Collegium Medicum, University of Warmia and Mazury in Olsztyn, 10-085 Olsztyn, Poland; t.antonowski@gmail.com (T.A.); adam.osowskiwnm@gmail.com (A.O.)

**Keywords:** inositol, liver, liver diseases, pharmacokinetics, hepatoprotective, *Danio rerio*, Wistar rats

## Abstract

Myo-inositol, the most common stereoisomer of inositol, plays an important role in many physiological processes, such as cell signaling, regulation of glucose and lipid metabolism, and protection of cells against oxidative stress. The main focus has been on pharmacokinetics, and it has been studied in both animal models (Wistar rats, mice, and *Danio rerio*) and humans. It is characterized by high oral bioavailability and is primarily eliminated via the kidneys. Preclinical studies have shown that myo-inositol has hepatoprotective potential, reducing oxidative stress, inflammation, and lipid accumulation in hepatocytes, as well as stabilizing liver cell membranes. Animal models make it possible to assess mechanisms of action, toxicity, and efficacy, thereby laying the groundwork for clinical research. In clinical practice, myo-inositol is currently used mainly in the treatment of polycystic ovary syndrome, gestational diabetes, fertility disorders, and certain affective disorders. Based on the results of preclinical studies, its potential application in liver diseases and drug-induced injury has been suggested. Despite promising findings, further translational research and randomized clinical trials are necessary to evaluate the therapeutic efficacy and safety of myo-inositol in hepatology. In summary, myo-inositol is a natural, well-tolerated compound with a multidirectional mechanism of action that may represent a promising element of supportive therapy for liver diseases.

## 1. Introduction

**Figure 1:**
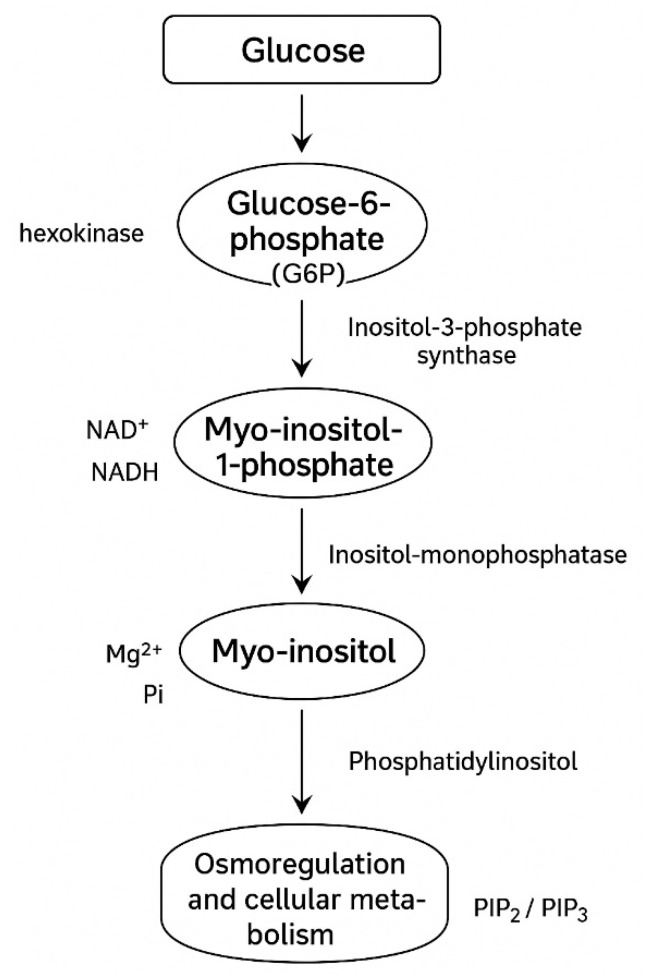
Biosynthesis pathway of myo-inositol in mammalian cells.

Cyclitols are a group of natural chemical compounds classified as polyols with a cyclic structure. Their common feature is a six-membered carbon ring in which each carbon atom is attached to a hydroxyl group. Among the nine stereoisomers of inositol, myo-inositol has the greatest biological significance, being the most widespread form in eukaryotic organisms. It is a key component of cell membranes and signaling metabolites, highlighting its role in the physiology and pathology of the organism [1,2] (Figure 1). Myo-inositol acts as a precursor of phosphoinositides and inositol trisphosphate (IP3), which are involved in cell signal transduction, including the regulation of calcium release from the endoplasmic reticulum. In this way, it participates in many cellular processes such as proliferation, apoptosis, differentiation, and transmembrane transport. Moreover, studies have indicated its significant role in regulating glucose and lipid metabolism, confirmed in polycystic ovary syndrome (PCOS) and insulin resistance therapy [3]. Myo-inositol also exhibits neuroprotective and antioxidant properties, which protect cells from oxidative stress. Such a wide range of activities make this compound a therapeutic candidate. In addition to its metabolic and regulatory functions, myo-inositol has been shown to influence mitochondrial activity and energetic homeostasis of the cell. Recent findings suggest that it contributes to the stabilization of mitochondrial membranes and enhances ATP production efficiency, thereby counteracting dysfunctions associated with metabolic syndrome and chronic liver injury [4]. Furthermore, its role in modulating adipokine secretion, such as adiponectin and leptin, links it directly to systemic metabolic regulation, including hepatic lipid turnover. This makes myo-inositol a compound of growing interest in hepatology, particularly in conditions characterized by lipotoxicity and insulin resistance [5,6].

**Figure 2:**
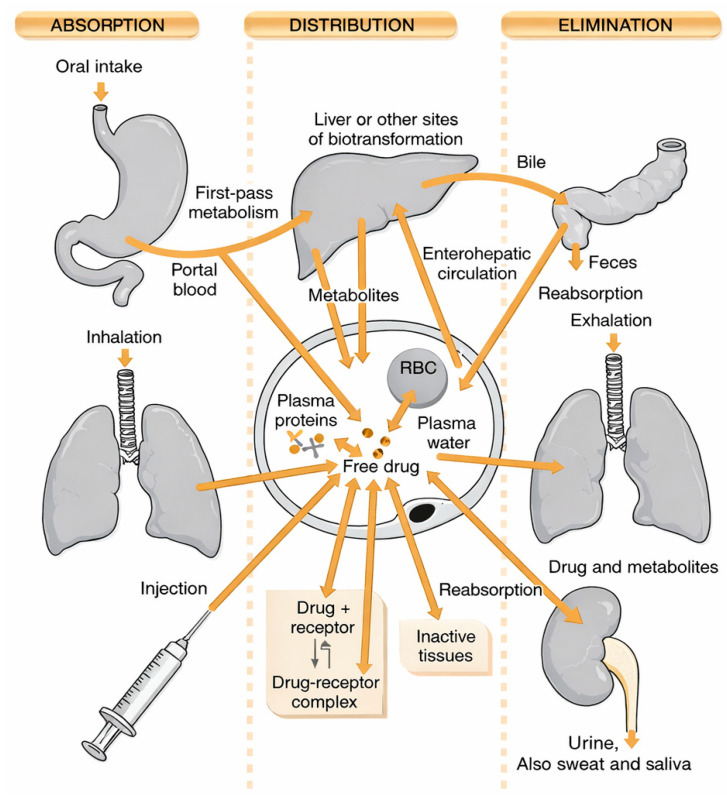
Pathways of drug absorption, distribution, and elimination in the body.

The aim of this paper is to integrate the available data concerning the pharmacokinetics of myo-inositol and its potential hepatoprotective effects. The analysis includes results from preclinical studies conducted on various animal models (e.g., Wistar rats, mice, zebrafish), as well as available clinical data [7]. This allows for a comparison of myo-inositol’s effects with other well-known hepatoprotectants, such as silymarin, N-acetylcysteine or curcumin. Preclinical studies represent an essential stage in the evaluation of the safety and efficacy of new biologically active substances. In the case of myo-inositol, whose safety profile has been well-documented (for example, in the treatment of PCOS and gestational diabetes), it is particularly important to expand knowledge about its potential in liver protection [8]. Animal models enable rapid and relatively cost-effective testing of hepatoprotective mechanisms, including the reduction in oxidative stress and the modulation of inflammatory responses. Importantly, studies highlight that myo-inositol supplementation can attenuate lipid peroxidation, normalize serum transaminase activity, and reduce histopathological signs of liver steatosis [9]. Comparative analyses with standard hepatoprotectants suggest that while its potency may not equal that of silymarin in acute toxin-induced damage, myo-inositol demonstrates superior efficacy in chronic metabolic disorders, where it directly targets insulin sensitivity and lipid dysregulation. In addition to oxidative stress and inflammation regulation, another critical mechanism underlying hepatoprotective activity is the MI modulation of gut–liver axis signaling (Figure 2).

Evidence indicates that myo-inositol may influence intestinal microbiota composition, enhancing populations of beneficial bacterial strains while reducing endotoxemia linked to non-alcoholic fatty liver disease [10]. This interaction between gut homeostasis and hepatic function further strengthens the rationality for exploring myo-inositol as a multifunctional therapeutic agent. The results of these studies may form the basis for future translational and clinical research aimed at developing new therapies to support the treatment of liver diseases, including non-alcoholic fatty liver disease (NAFLD) and non-alcoholic steatohepatitis conditions (NASH) of growing epidemiological significance. In particular, including myo-inositol into combination therapies, alongside antioxidants, insulin sensitizers, or microbiota-targeted interventions, may represent a novel approach to comprehensive liver protection. Given the global burden of chronic liver diseases, the exploration of such safe and well-tolerated compounds offers a promising perspective for preventive and therapeutic strategies in modern hepatology [11].

Myo-inositol belongs to a highly dynamic and biologically influential family of inositol phosphates, a group of molecules that function as central regulators of cellular signaling, metabolism, and redox balance. While myo-inositol itself serves as the structural backbone, its phosphorylated derivatives—collectively known as inositol phosphates (IPs)—are responsible for many of the most significant biological effects attributed to this family [12]. Among the lower phosphorylated forms, inositol monophosphate (IP1) and inositol bisphosphate (IP2) act primarily as intermediates within the phosphoinositide signaling cycle. Of particular importance is inositol trisphosphate (IP3), a pivotal second messenger generated from membrane phosphatidylinositol 4,5-bisphosphate (PIP2). IP3 regulates intracellular calcium release from the endoplasmic reticulum, thereby controlling processes such as muscle contraction, hormone secretion, neurotransmission, and immune cell activation. Subsequent phosphorylated derivatives, including inositol tetrakisphosphate (IP4) and inositol pentakisphosphate (IP5), further refine calcium signaling and participate in nuclear functions such as RNA export, chromatin remodeling, and DNA repair [13]. Special attention should be given to inositol hexakisphosphate (IP6), also known as phytic acid. IP6 has been extensively investigated for decades and is recognized for its antioxidant properties, largely due to its capacity to chelate excess iron and thereby limit hydroxyl radical formation. Beyond its redox activity, IP6 has demonstrated multiple biological effects relevant to human health, including modulation of cell proliferation and differentiation, induction of apoptosis, inhibition of angiogenesis, support of immune function, and potential roles in metabolic regulation and kidney stone prevention [14]. Its documented involvement in both disease prevention and adjunctive therapeutic strategies underscores its importance within the inositol phosphate family. Collectively, the inositol phosphate network represents an integrated signaling system that extends far beyond the activity of myo-inositol alone. The diverse biological functions of these related molecules—spanning calcium signaling, gene regulation, antioxidant defense, metabolic control, and cellular growth pathways—illustrate the complexity and therapeutic potential of this biochemical family [15].

## 2. Biology and Physiology of Myo-Inositol

Inositol is a hexahydroxycyclohexane whose unique stereochemistry allows the formation of nine stereoisomers. The most widespread biological form is myo-inositol, whose spatial configuration favors its role in the formation of phosphatidylinositols and phosphoinositides—key components of cell membranes and mediators of intracellular signaling [11]. Myo-inositol can be synthesized endogenously in the liver and kidneys through the conversion of glucose-6-phosphate via the action of the enzyme inositol-3-phosphate synthase Figure 1. Despite this biosynthetic capacity, a significant portion of the body’s requirement is also supplied by the diet-rich sources, including fruits, whole grains, legumes, and nuts. Clinical supplementation of myo-inositol, for instance in PCOS, represents an additional exogenous source, enabling its higher serum and tissue concentrations. Myo-inositol is transported into cells primarily through specific sodium-dependent transporters (SMIT1 and SMIT2), as well as the proton-dependent transporter HMIT [16]. Their expression is tightly regulated by extracellular osmolarity, allowing cells to maintain proper intracellular MI levels under stress conditions, such as hyperglycemia or hyperosmolarity. Once inside the cell, myo-inositol distribution is compartmentalized between the cytosol, endoplasmic reticulum, and plasma membrane domains, reflecting its role as a substrate for the synthesis of phosphatidylinositols and other derivatives involved in signaling processes. The catabolism of myo-inositol occurs mainly in the kidneys, where it is degraded into carbon dioxide and water, highlighting its dynamic metabolic turnover. Importantly, impaired inositol metabolism has been linked to pathological states such as diabetic nephropathy, where increased urinary inositol excretion contributes to tissue depletion [17] (Figure 2; Table 1).

## 3. Pharmacokinetics of Myo-Inositol

**Figure 3:**
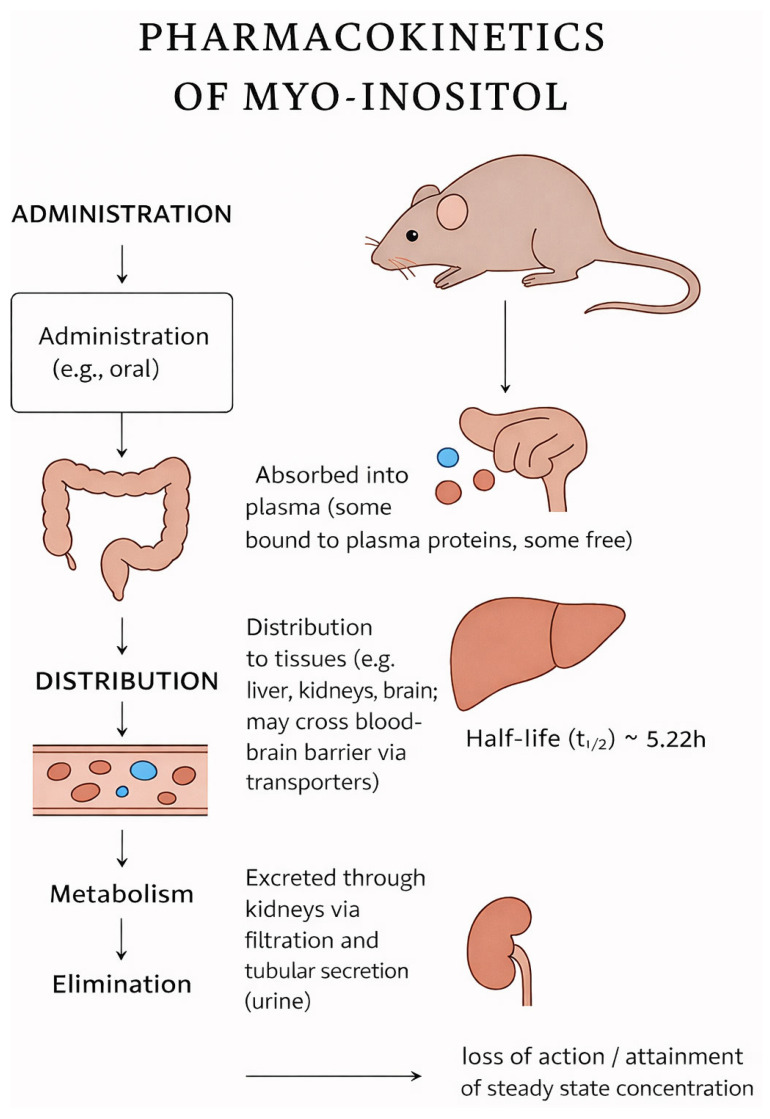
Pharmacokinetics of myo-Inositol.

Preclinical studies have provided important insights into the pharmacokinetics of myo-inositol. In animal models such as Wistar rats, mice, and zebrafish (*Danio rerio*), it has been demonstrated that the bioavailability and metabolism of this compound depend on the route of administration. Following oral administration, intestinal absorption of myo-inositol is relatively rapid and occurs mainly via SMIT1/2 transporters, which are sodium-dependent inositol symporters responsible for maintaining cellular osmoregulation and phosphoinositide synthesis [8,9,20]. In addition, evidence has suggested that HMIT (H+/myo-inositol transporter) localized in the central nervous system may also contribute to tissue-specific uptake, particularly in the brain. Studies on rats has revealed that the highest plasma concentrations were observed within 1–2 h after oral intake, while the half-life ranged from 4 to 8 h. In the case of intravenous administration, higher bioavailability and faster tissue distribution were achieved, though the primary route of elimination remains glomerular filtration and urinary excretion. It should be noted that the intravenous administration of myo-inositol is rare and only employed within specific situations. In zebrafish, myo-inositol supplementation was associated with improved metabolic parameters and protection of hepatocytes against oxidative stress, which indirectly indicates its active hepatic metabolism [8] (Figure 3).

Clinical studies in humans have shown that the pharmacokinetic profile of myo-inositol is similar to that observed in rats. Following oral supplementation (2–4 g/day), stable plasma concentrations are reached within a few days, and the highest concentration after a single dose occurs within 1–3 h. Elimination takes place primarily via the kidneys, as in experimental animals. The main differences concern the rate of hepatic metabolism-degradation. The processes are slower in humans, which may contribute to the longer persistence of higher tissue concentrations [8]. This prolonged tissue availability may be of clinical relevance, particularly in the liver and ovaries, where myo-inositol is implicated in regulating glucose uptake, insulin sensitivity, and steroidogenesis. Myo-inositol, as a natural component of the body, is relatively safe and well-tolerated. However, there are reports suggesting that its bioavailability and pharmacokinetics may change in the presence of certain drugs. For example, the use of lithium in psychiatric therapy may affect myo-inositol metabolism by inhibiting the phosphoinositide pathway and lowering inositol concentrations in the brain. This observation has been mechanistically linked to the therapeutic action of lithium in mood disorders, as reduced inositol turnover can modulate signaling cascades related to neurotransmitter function. Conversely, myo-inositol supplementation may potentially synergize with antioxidants (e.g., vitamin E, curcumin), enhancing their protective effects on the liver and other organs exposed to oxidative stress [7]. There is also preliminary evidence suggesting that co-administration with D-chiro-inositol and alpha-lipoic acid may improve insulin resistance and reproductive outcomes in patients with polycystic ovary syndrome (PCOS), further highlighting the importance of pharmacodynamic interactions.

## 4. Myo-Inositol as a Potential Hepatoprotective Compound

Myo-inositol may exert hepatoprotective effects through several biological mechanisms. A key role is played by its involvement in lipid metabolism regulation, modulation of oxidative stress, and influence on insulin signaling. Animal model studies have shown that myo-inositol supplementation reduces triglyceride accumulation in hepatocytes, which is associated with improved insulin sensitivity and reduced lipotoxicity. Moreover, myo-inositol and its derivatives (including D-chiro-inositol) affect the activation of antioxidant enzymes such as superoxide dismutase (SOD) and catalase, thereby limiting oxidative damage to hepatocytes [7,21]. Experiments conducted on hepatocyte cell lines confirmed that myo-inositol reduces oxidative stress induced by hydrogen peroxide and ethanol. In vitro studies have also demonstrated its ability to inhibit the expression of apoptosis markers such as caspase-3, as well as to enhance cell viability under conditions of metabolic stress. Importantly, this effect was dose-dependent, with optimal protective effects observed at concentrations close to physiological levels. In animal models of NAFLD, myo-inositol supplementation led to a reduction in hepatic fat content, improvement in lipid profile, and decreased activity of transaminases (ALT, AST). In models of toxic liver injury (e.g., induced by CCl_4_), myo-inositol was shown to reduce lipid peroxidation and restore antioxidant balance in liver tissue. These findings suggest that myo-inositol exerts protective effects in both metabolic and toxic hepatocyte damage. Additionally, there is evidence that myo-inositol participates in the regulation of mitochondrial function, supporting ATP production and reducing the generation of reactive oxygen species (ROS), which further contributes to the maintenance of hepatocyte integrity [22,23]. Beyond its direct antioxidant and metabolic actions, myo-inositol may also influence inflammatory pathways within the liver. Preclinical studies have indicated that supplementation with inositol can attenuate the expression of pro-inflammatory cytokines such as TNF-α and IL-6, which play a pivotal role in the progression of NAFLD to NASH. By modulating these signaling cascades, myo-inositol may help prevent fibrosis and subsequent liver dysfunction. Furthermore, its impact on phosphatidylinositol signaling pathways suggests a broader role in cell membrane stability and hepatocyte regeneration after injury [23]. Clinical data regarding the hepatoprotective effects of myo-inositol are limited; however, there is evidence that supplementation may be beneficial in patients with NAFLD and metabolic syndrome. Pilot studies have shown that administration of myo-inositol to patients with obesity and insulin resistance resulted in decreased liver enzyme levels and improved metabolic markers [24]. Other preliminary trials suggest that myo-inositol, when administered in combination with D-chiro-inositol, may enhance glycemic control, reduce visceral adiposity, and improve hepatic function parameters. Despite these promising observations, large-scale randomized controlled trials are still needed to establish standardized dosing regimens, long-term efficacy, and potential synergistic effects with other therapeutic strategies. Given its good tolerance and safety profile, myo-inositol may represent a promising adjunctive therapy in liver diseases, particularly when combined with other dietary and pharmacological interventions [25]. Its pleiotropic effects on oxidative stress, inflammation, and lipid metabolism position it as a potential candidate not only for NAFLD but also for other conditions associated with hepatic injury, including drug-induced hepatotoxicity and viral hepatitis. In the most recent work by Antonowski and colleagues, a comprehensive analysis of the pharmacokinetics of myo-inositol in various animal models was presented [7]. The authors confirmed its good bioavailability after oral administration, rapid tissue distribution, and the dominant role of the kidneys in its elimination. Importantly, they demonstrated that hepatic metabolism of myo-inositol plays a significant role in modulating oxidative stress and may represent a key element of its hepatoprotective mechanism [8]. Beyond hepatic protection, emerging data suggest that myo-inositol might influence mitochondrial function and cellular redox balance, providing an additional explanation for its broad therapeutic potential. Taken together, these findings support the concept of using myo-inositol as a potential candidate for further translational research in the context of liver disease therapies. At the same time, its favorable pharmacokinetic profile, combined with excellent tolerability, strengthens its position as an attractive adjunctive compound in metabolic, neurological, and reproductive disorders [21]. Future research should focus on elucidating molecular targets of myo-inositol in hepatocytes, identifying patient populations most likely to benefit, and integrating supplementation strategies into comprehensive treatment protocols for chronic liver diseases [25,26].

## 5. Animal Models for Investigating the Hepatoprotective Effects of Myo-Inositol

**Figure 4:**
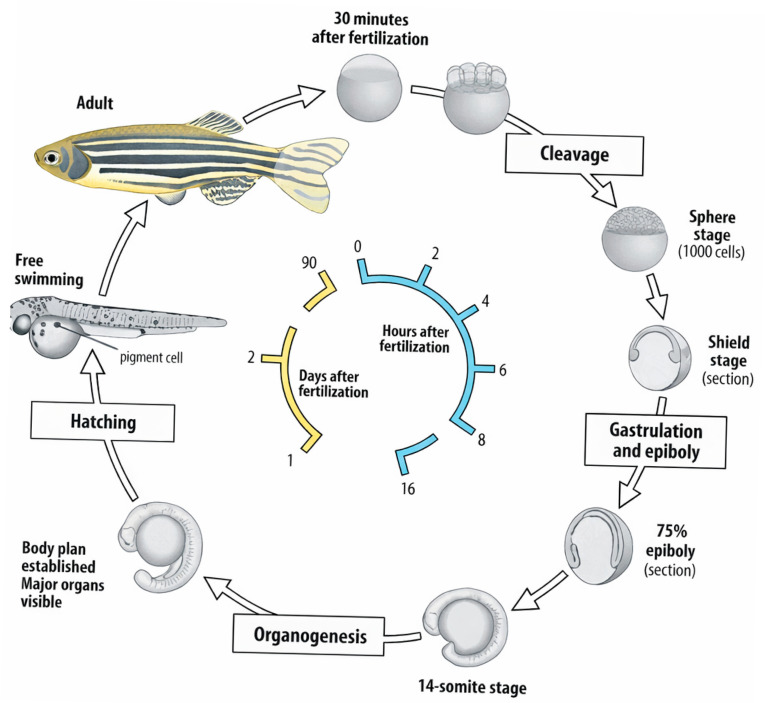
Life cycle of the zebrafish.

Wistar rats are among the most commonly used models in preclinical studies on the metabolism and pharmacokinetics of myo-inositol. Their main advantages include a well-characterized metabolic profile, reproducibility of results, and the ability to precisely control experimental conditions, such as diet, circadian rhythm, and exposure to hepatotoxic agents [1,27]. Studies in rats have shown that myo-inositol supplementation improves metabolic parameters, reduces lipid accumulation in hepatocytes, lowers liver enzyme levels, and enhances antioxidant defense through modulation of glutathione and superoxide dismutase activity. Furthermore, long-term administration has been associated with reduced insulin resistance and improved lipid metabolism, which is particularly relevant in NAFLD models. However, a limitation is the difference in hepatic metabolic rate and phase I/II biotransformation enzymes between rats and humans, which may affect the translational value of the results [8]. The zebrafish (*Danio rerio*) is gaining increasing importance in studies of the toxicity and therapeutic potential of myo-inositol. Due to the transparency of larval bodies, direct observation of metabolic processes, angiogenesis, and organ damage is possible using advanced imaging techniques. In models of hepatotoxicity induced by alcohol or CCl_4_, it has been shown that myo-inositol supplementation protects hepatocytes from oxidative stress, improves mitochondrial function, and enhances overall survival [28]. In addition, zebrafish models enable high-throughput screening of dose–response relationships and assessment of developmental toxicity, which is difficult to achieve in mammalian systems. The short life cycle, genetic similarity to humans, and ease of CRISPR/Cas9-based modifications make *Danio rerio* a valuable tool in screening, mechanistic studies, and gene–nutrient interaction analyses [7,28] (Figure 4).

Both rodent and zebrafish models provide important data on the safety and potential hepatoprotective mechanisms of myo-inositol [7,8,9]. In the case of rats, the results are particularly valuable for pharmacokinetic and metabolic studies, including tissue distribution, renal clearance, and enterohepatic circulation [8,9]. Meanwhile, zebrafish enables rapid testing of toxicity, protective effects, and genetic pathways involved in hepatoprotection under high-throughput conditions [7,29]. Importantly, these complementary models allow researchers to identify both molecular mechanisms (e.g., modulation of PI3K/Akt signaling, reduction in lipid peroxidation) and systemic outcomes (e.g., normalization of liver enzymes, improved survival rates). Translating these findings into clinical studies, however, requires caution due to interspecies differences in metabolism, bioavailability, microbiota-dependent inositol degradation, and sensitivity to oxidative stress [30]. The validation process for the hepatoprotective effects of myo-inositol should include several stages:

1. Screening studies on simple models, such as *Danio rerio*, to determine basic mechanisms of action, identify potential off-target effects, and establish toxicity thresholds.
2. Studies on rodent models, enabling detailed analysis of pharmacokinetics, metabolism, tissue distribution, and therapeutic efficacy under conditions closer to clinical settings.
3. Comparison with reference drugs (e.g., silymarin, N-acetylcysteine), which places myo-inositol in the context of available hepatoprotective therapies and helps to evaluate its potential advantages or synergistic effects.
4. Translational studies, including large animal models (e.g., pigs, dogs) and early clinical trials, to assess safety, optimal dosing, and efficacy in humans.

In summary, animal models constitute an indispensable stage in myo-inositol research, allowing for both the evaluation of hepatoprotective mechanisms and the preparation of groundwork for future clinical studies. The integration of data from zebrafish, rodents, and larger mammals provides a robust preclinical framework, while comparative studies with standard therapies ensure that the clinical relevance of myo-inositol is critically evaluated before its translation into human medicine [31].

## 6. Clinical Implications and Future Directions

Myo-inositol is already widely used in clinical practice today, mainly as a dietary supplement. The best-documented areas of application include polycystic ovary syndrome (PCOS), where it improves insulin sensitivity, restores menstrual cycle regularity, and increases ovulation frequency. It is also used as an adjunct therapy in insulin resistance, gestational diabetes, and fertility disorders [32]. Moreover, a beneficial effect has been observed in depression and affective disorders, which is linked to the modulation of the phosphoinositide pathway in the brain. Beyond these indications, myo-inositol has been studied in the context of metabolic syndrome and obesity, where it has demonstrated potential to improve glucose tolerance, lipid profile, and reduce markers of systemic inflammation. Clinical trials suggest that supplementation may contribute to lowering triglyceride levels and improving the HDL/LDL balance, which further supports its role in preventing cardiovascular complications associated with insulin resistance and liver dysfunction. Based on preclinical studies, myo-inositol shows hepatoprotective potential. In animal models, it has been demonstrated to reduce oxidative stress, decrease inflammation, and stabilize hepatocyte cell membranes [7,33]. These mechanisms of action suggest its usefulness in the treatment of conditions such as NAFLD and its advanced form, NASH, as well as in liver injuries induced by drugs or toxins. Additional experimental data indicate that myo-inositol supplementation can influence mitochondrial function by improving ATP synthesis and reducing lipid peroxidation, which are crucial factors in preventing hepatocyte apoptosis and fibrosis progression [34]. From a mechanistic perspective, the hepatoprotective effects of myo-inositol are attributed not only to its antioxidant properties but also to the regulation of lipid metabolism through the activation of AMP-activated protein kinase (AMPK) and downregulation of sterol regulatory element-binding proteins (SREBPs). These molecular pathways are directly implicated in hepatic steatosis and represent attractive therapeutic targets. Currently, myo-inositol is available in most countries as a dietary supplement rather than a registered drug. This means its use does not require undergoing the full clinical trial procedure, and the evidence for efficacy and safety remains limited to observational studies and small-scale interventions [35]. These barriers may delay the broader adoption of myo-inositol as a hepatoprotective therapy, particularly in the context of chronic liver diseases. An additional challenge is the lack of standardized dosing regimens and the variability in bioavailability depending on the formulation used. While doses ranging from 2 to 4 g daily are commonly applied in PCOS and metabolic disorders, optimal dosing for hepatoprotection has not been clearly defined. Moreover, it is not yet established whether myo-inositol should be used as monotherapy or in combination with other hepatoprotective agents for synergistic effects [7,8]. Existing data indicate significant potential of myo-inositol as a hepatoprotective compound; however, large-scale randomized clinical trials are necessary to assess its effectiveness in patients with NAFLD, NASH, or drug-induced liver injury. Particularly important is the comparison of its effects with other well-known hepatoprotectants, such as silymarin, N-acetylcysteine, or curcumin. Future studies should also consider evaluating interactions between myo-inositol and drugs commonly used chronically in hepatological patients [8,36]. Furthermore, precise medical approaches may help identify subgroups of patients—such as those with concomitant insulin resistance or specific genetic polymorphisms in inositol metabolism—who could benefit most from supplementation. In summary, myo-inositol represents a promising candidate for expanding clinical applications in hepatology; however, its path to becoming a fully recognized therapy requires further translational and clinical research. If ongoing studies confirm its efficacy and safety, myo-inositol could become not only an adjunct but also a first-line therapeutic option in the management of metabolic and liver disorders [36].

## 7. Conclusions

Myo-inositol, the predominant stereoisomer of inositol, plays a pivotal role in cell signaling, glucose and lipid metabolism, osmoregulation, and neuroprotection, largely through its role as a precursor of secondary messengers such as inositol triphosphates (IP3). Preclinical studies demonstrate high oral bioavailability and primarily renal elimination. In animal models, myo-inositol exerts hepatoprotective effects by reducing oxidative stress, stabilizing hepatocyte membranes, enhancing mitochondrial function, modulating inflammatory cytokines, improving insulin sensitivity, reducing lipid accumulation, and attenuating fibrogenesis [7,37]. The rising prevalence of NAFLD and NASH, along with the clinical burden of drug-induced liver injury, underscores the need for effective and safe hepatoprotective agents. Current therapeutic options remain limited, with lifestyle interventions as the mainstay and pharmacological treatments often yielding suboptimal outcomes or adverse effects. Myo-inositol’s favorable safety profile and its demonstrated metabolic benefits in other clinical contexts, such as polycystic ovary syndrome and gestational diabetes, highlight its translational potential in hepatology [38]. Despite promising preclinical data, robust clinical evidence is lacking. Future research priorities include translational studies in large animal models, development of 3D in vitro liver systems and organoids to elucidate cellular mechanisms, evaluation of interactions with hepatotoxic and metabolic drugs, phase II/III clinical trials in NAFLD, NASH, and drug-induced liver injury, and biomarker identification to enable personalized therapy [8,39,40].

In summary, myo-inositol represents a promising hepatoprotective candidate. Its safety, multifaceted mechanisms, and translational potential warrant further investigation, and, if clinically validated, it could complement current strategies for managing metabolic liver diseases [41].

## Figures and Tables

**Table 1:** MI role in physiological processes.

Physiological Process	Scientific Description
Cell signaling	Key element in the production of phosphatidylinositol and its phosphorylated forms (PIP, PIP2, PIP3), which participate in signaling cascades such as the PI3K/Akt and PLC-IP3/DAG pathways Influencing cell proliferation, metabolism, and survival Derivatives such as inositol polyphosphates (IP3, IP4, IP6) act as secondary messengers regulating nuclear processes, chromatin remodeling, and vesicle trafficking [10]
Regulation of lipid and glucose metabolism	Improve insulin signaling through modulation of the PI3K/Akt pathway Supports improved insulin sensitivity and reduction in dyslipidemia parameters Specific isomer D-chiro-inositol functions as insulin sensitizer by enhancing glycogen synthesis [3,11]
Neuroprotective and antioxidant effects	Exhibits the ability to protect neural cells by reducing oxidative stress and stabilizing mitochondrial membranes Potential biomarker of neuroinflammation in conditions such as multiple sclerosis or hepatic encephalopathy Neurodegenerative diseases such as Alzheimer’s disease, where it may modulate phosphoinositide metabolism and calcium homeostasis [18]
Reproductive health	Regulates follicular maturation and oocyte quality, which is particularly relevant in infertility associated with PCOS Improving ovulation rates, hormonal balance, and overall fertility outcomes Role in male fertility, where supplementation enhances sperm motility and mitochondrial function [3,11,19]
Osmoregulation	Serves as an essential organic osmolyte, allowing cells to adapt to hyperosmotic stress, especially in the brain, kidney medulla, and lens of the eye [6]

## Data Availability

No new data were created or analyzed in this study. Data sharing is not applicable to this article.
